# Assessment of fatigability in patients with spinal muscular atrophy: development and content validity of a set of endurance tests

**DOI:** 10.1186/s12883-019-1244-3

**Published:** 2019-02-09

**Authors:** Bart Bartels, Laura E. Habets, Marloes Stam, Renske I. Wadman, Camiel A. Wijngaarde, Marja A. G. C. Schoenmakers, Tim Takken, Erik H.J. Hulzebos, W. Ludo van der Pol, Janke F. de Groot

**Affiliations:** 1 Child Development and Exercise Center, Wilhelmina Children’s Hospital, University Medical Center Utrecht, PO Box 85090, KB 02.056.0, 3508 AB Utrecht, The Netherlands; 2 Department of Neurology and Neurosurgery, Brain Center Rudolf Magnus, University Medical Center Utrecht, Utrecht, The Netherlands; 3 Netherlands Institute for Health Services Research (Nivel), Utrecht, The Netherlands

**Keywords:** Spinal muscular atrophy, Fatigability, Endurance, Outcome measure

## Abstract

**Background:**

Fatigability has emerged as an important dimension of physical impairment in patients with Spinal Muscular Atrophy (SMA). At present reliable and valid outcome measures for both mildly and severely affected patients are lacking. Therefore the primary aim of this study is the development of clinical outcome measures for fatigability in patients with SMA across the range of severity.

**Methods:**

We developed a set of endurance tests using five methodological steps as recommended by the ‘COnsensus-based Standards for the selection of health Measurement INstruments (COSMIN). In this iterative process, data from multiple sources were triangulated including a scoping review of scientific literature, input from a scientific and clinical multidisciplinary expert panel and three pilot studies including healthy persons (*N* = 9), paediatric patients with chronic disorders (*N* = 10) and patients with SMA (*N* = 15).

**Results:**

Fatigability in SMA was operationalised as the decline in physical performance. The following test criteria were established; one method of testing for patients with SMA type 2–4, a set of outcome measures that mimic daily life activities, a submaximal test protocol of repetitive activities over a longer period; external regulation of pace. The scoping review did not generate suitable outcome measures.

We therefore adapted the Endurance Shuttle Walk Test for ambulatory patients and developed the Endurance Shuttle *Box and Block Test* and the - *Nine Hole Peg Test* for fatigability testing of proximal and distal arm function. Content validity was established through input from experts and patients. Pilot testing showed that the set of endurance tests are comprehensible, feasible and meet all predefined test criteria.

**Conclusions:**

The development of this comprehensive set of endurance tests is a pivotal step to address fatigability in patients with SMA.

**Electronic supplementary material:**

The online version of this article (10.1186/s12883-019-1244-3) contains supplementary material, which is available to authorized users.

## Background

Hereditary proximal Spinal Muscular Atrophy (SMA) is an autosomal recessive neurodegenerative disease caused by homozygous loss of function of the survival motor neuron 1 (*SMN1*) gene [1]. SMA is characterised by a wide range of disease severity ranging from neonatal respiratory insufficiency and death (SMA type 1), ability to sit without support but inability to walk independently (SMA type 2), problems with or the loss of ambulation (SMA type 3a-b) to relatively mild impairments due to proximal muscle weakness in patients with adult onset disease (SMA type 4) [2]. All four SMA types are characterised by progressive muscle weakness and secondary loss of motor abilities over time [3]. In addition to muscle weakness, fatigability has emerged as a rather common but often overlooked complaint among patients with SMA [4, 5]. The current taxonomy defines ‘fatigability’ as the magnitude or rate of change in a performance criterion relative to a reference over a given time of task performance or measure of mechanical output and is the opposite of ´endurance´, which involves the prolonged maintenance of constant or self-regulated power or velocity [6, 7]. Patients with SMA refer that they easily fatigue during repetitive activities of daily living such as lifting an arm during eating or walking even short distances. A possible explanation comes from SMA animal models and post-mortem studies that showed abnormal development and maturation of the neuromuscular junction. Neuromuscular dysfunction has been found in at least half of the patients with SMA, suggesting that this may contribute to complaints of fatigability [5, 8–12]. Since outcome measures sensitive to change in fatigability are lacking, their development is a pivotal step in a better understandig of fatigabillity in SMA [13, 14]. This study aimed to provide the framework for the development of novel clinical outcome measures for fatigability in patients with SMA type across the range of severity. We determined content validity following the COnsensus-based Standards for the selection of health Measurement INstruments (COSMIN) -guidelines and recommendations by European and American regulatory authorities [15, 16].

## Methods

A set of outcome measures for fatigability was developed according to five methodological steps as recommended by COSMIN (Table 1) [17, 18]. In this iterative process data from multiple sources were triangulated. Sources included a scoping review of scientific literature, input from a scientific and clinical multidisciplinary expert panel and three pilot studies including healthy persons (*N* = 9), paediatric patients with chronic disorders (*N* = 10) and patients with SMA (*N* = 15). The expert panel consisted of ten clinicians and researchers including paediatric physical therapists (BB, MS), clinical exercise physiologists and movement scientists (JG, LH, HH,TT) and neurologists or neurology residents with ample experience in caring for children and adults with SMA (MS, CW, RW, WP). Three round table discussions took place with different group compositions.

**Table 1:** Methodological steps COSMIN^a^

Methodological steps	Questions to be answered	Sources
Step 1: Definition and elaboration of the construct	1) Definition of fatigability? 2) Target population? 3) Purpose of the outcome measure?	• Key papers on fatigability assessment
Step 2: Choice of measurement method	1) Existing measurement that responds closely to construct to be measured? 2) Level of measurement? 3) Single or multiple measures?	• Scoping review of scientific literature • Expert panel (round table discussion 1)
Step 3: Selecting and formulating items	1) Which activities cause most problems? 2) Which available measures reflect these activities?	• Patient report outcome (pilot sample 3) • Expert panel (round table discussion 2)
Step 4: Scoring issues	1) Application in research or clinical practice? 2) Measurement level?	• Expert panel (round table discussion 3)
Step 5: Pilot testing	1) Comprehensibility? 2) Feasibility? 3) Relevance?	• Pilot sample 1 (healthy subjects) • Pilot sample 2 (pediatric patients with chronic diseases) • Pilot sample 3 (patients with SMA)

^a^COSMIN = ‘COnsensus-based Standards for the selection of health Measurement Instruments

### Definition and elaboration of the construct intended to be measured

The first step in the development of a new outcome measure consisted of the operationalization of the theoretical construct in SMA. This included a clear definition of fatigability, a description of the target population and the purpose of the outcome measure and the composition of specific test criteria. The taxonomy for fatigue and fatigability as proposed by Kluger et al. was used as a starting point from which a construct for fatigability assessment in SMA was described. Fatigability was defined as the magnitude or rate of change in a performance criterion relative to a reference over a given time of task performance or measure of mechanical output [7]. Several other key papers on fatigability that used a similar definition and described test methodology were selected to complement the framework [19–25].

### Choice of measurement method

During the second step we combined the results from a scoping review on available measures for fatigability in patients with SMA with the experiences with fatigability testing by our research group.

### Scoping review of the literature

Given the fact that SMA has been associated with fatigability only recently, it was anticipated that a systematic review would not generate significantly more information than a scoping literature search. Peer-reviewed experimental articles written in English were retrieved from Pubmed and Trial.gov up to the first of October 2014. The following search strings was used*: ((“muscular atrophy, spinal”[MeSH Terms] OR (“muscular”[All Fields] AND “atrophy”[All Fields] AND “spinal”[All Fields]) OR “spinal muscular atrophy”[All Fields] OR (“spinal”[All Fields] AND “muscular”[All Fields] AND “atrophy”[All Fields])) OR (“Stat Methods Appt”[Journal] OR “sma”[All Fields])) AND (((Fatigability [All Fields] OR Endurance [All Fields]) OR Stamina [All Fields]) OR (“fatigue”[MeSH Terms] OR “fatigue”[All Fields])).* At first, papers were selected that described the measurement of fatigability or endurance in patients with SMA. Secondly, outcome measures were assessed to what extent they complied with the definition and test criteria defined within this study. In the case that no suitable outcome measure were retrieved, the expert panel discussed in the first round table discussion whether other appropriate outcome measure were available that met the clinimetric requirements and could be validated for SMA.

### Selecting and formulating items

During the third step, questionnaires were taken from the pilot sample of patients with SMA to determine which activities of daily living (ADLs) provoked fatigability. In adults, the questionnaire by Straver et al. was used which was originally validated for peripheral nervous system disorders [26]. A similar questionnaire was developed for children based on clinical experience from the expert panel and items from the Child Health Assessment Questionnaire, a validated questionnaire for ADLs in other clinical populations [27]. Patient-reported activities that caused fatigability were clustered into three different functional domains, namely leg function, upper arm function and hand function. The expert panel assessed in the second round table discussion whether all domains were relevant to SMA and should be included in the development of the set of outcome measures for fatigability.

### Scoring issues

During the fourth step, the expert panel discussed about the composition of the tests, taking into account the application setting (research, clinical practise) and the patient group, and selecting primary outcome parameters. For example, tests are usually shorter in clinical practise, due to time constraints [17].

### Pilot testing

Patients with SMA were recruited from the Dutch SMA registry (http://www.treat-nmd.eu/registry/310/) [28]. This registry contains detailed clinical information of over 300 children and adults with SMA. To minimize selection bias, all eligible patients listed in this register were offered the possibility to participate. All patients had a confirmed homozygous deletion of the *SMN1* gene or a heterozygous *SMN1* deletion in combination with a point mutation on the second *SMN1* allele. In order to be eligible to participate in this study, a subject had to meet all of the following additional criteria: age 8–60 years; ability to follow test instructions and no exercise restrictions. Two patients with SMA declined participation due to frequent hospital visits in the recent past and fear of increased fatigue. Patient controls were recruited from a school for special education in Utrecht. Healthy controls were recruited from the University of Applied Sciences and the University Medical Center Utrecht. The outcome measures for fatigability were pilot-tested on *‘comprehensibility’* (‘Are test instructions to participants unambiguous and well understood?’) and *‘feasibility’* (measurement completion, acceptability and perceived burden) in three consecutive pilot samples of healthy controls (pilot sample 1), paediatric patients with chronic diseases (pilot sample 2) and patients with SMA (pilot sample 3). *‘*Measurement completion rate’ was defined as the number of participants able to complete the test without premature discontinuation caused by motivational issues or a-specific physical complaints [17]. ‘Acceptability’ was defined as the willingness to perform the test again in the future and was assessed with a ‘Visual Analogue Scale’ [29]. Perceived burden was assessed with the OMNI scale for perceived exertion [30]. The third round table discussion was used to discuss pilot data and if necessary to make small adjustments to the protocol.

## Results

### Definition and elaboration of the construct intended to be measured

Fatigability is subdivided in ´physical fatigability’ and ‘cognitive fatigability’ which are measured in different ways [7, 21, 24, 31]. ‘Physical fatigability is primarily measured by quantifying the decline in one or more aspects of motor performance such as peak force, power, speed and accuracy while cognitive fatigability is measured by quantifying the decline in processing speed and sustained attention over time during a sustained complex information processing task. Given the fact that patients with SMA complain about sustaining physical activities, we decided to focus on physical fatigability defined as a decline in performance such as peak force, power, speed and accuracy. A number of methods have been described to measure fatigability during different types of performances including:

1. Continuous performance of a prolonged task [19, 21]:

- Intermitted submaximal exercise protocol which mimics activities such as walking or cycling in which fatigability develops over a longer period
- Continuous maximal protocol which mimics activities such as lifting heavy objects or sprinting

2) Comparing performance on a probe task before and immediately after prolonged performance of a separate fatigue inducing task [32].

Fatigability is experienced by patients with SMA as the inability to perform prolonged repetitive tasks during activities of daily life. These complaints are reminiscent of those of patients with myasthenic syndromes, which are caused by reduced efficiency of neuromuscular junction [33]. Moreover, SMA is characterised by structural and physiological abnormalities of the neuromuscular junction as shown by post-mortem studies and the presence of pathological decrement upon repetitive nerve stimulation supporting the hypothesis that neuromuscular junction dysfunction is associated with fatigability in SMA and should be the focus of fatigability test development. The extent of fatigability may vary according to the method of testing [22, 24]. Therefore, test protocols should be used that mimic daily life activities that provoke fatigability in patients. Consequently a set of predefined test-criteria were composed (Table 2).

**Table 2:** Test criteria for SMA and candidate outcome measures

	Methodology	Type	Protocol	Standardization	Intensity	Test duration	External regulation of pace
Pre-defined test criteria	Generic applicable	Mimic daily life activities	Repetitive tasks	Yes	Submaximal	➢ 75 s*	yes
RNHPT	+/−	+	+	+	+	+	–
Sustained MVC during 60 s	–	–	–	+	–	+	–
Sustained MVC during 15 s	+	+/−	–	+	–	–	–
Masticatory function	**–**	–	–	+	–	–	**–**
6MWT	**–**	+	+	+/−	+	+	–
ESWT	**–**	+	+	+	+	+	+

*RNHPT* = Repeated Nine Hole Peg Test, *MVC* = Maximal Voluntary Contraction, *6MWT* = 6 Minute Walk Test, *ESWT* = Endurance Shuttle Walk Test, *Mn* = Mean value, *Gastin et al. 2010 [63]

### Choice of measurement method

The scoping review search performed on the 1st of October 2014 retrieved 109 records in Pubmed and no additional records in trial.gov. All records were screened on title and abstract. Seven papers were included describing 4 different methods to assess fatigability in SMA (Additional file 1): Sustained maximal voluntary contraction for 60 s [34], Sustained maximal voluntary contraction for 15 s [35], Masticatory endurance [36] and the Six Minute Walk Test (6MWT) [37–40]. We recently reported our experience with the *repeated Nine Hole Peg Test* (rNHPT) as a measure for fatigability of arm and hand function in patients with SMA. Given the promising results, the r9HPT was included in the assessment of potential outcome measures derived from the review. Recently, this study was published [41].

### Evaluation of selected outcome measures

All five different outcome measures from the methods above, defined fatigability as the decrease in physical performance, which was in accordance with the definition used in this study. There was however a large difference in methods of testing with regards to target muscles, type of exercise, intensity and duration (Table 2). The rNHPT and the 6MWT, both submaximal repetitive tasks, met most predefined criteria and provided proof of principle that including an endurance element holds promise as a mode to measure fatigability objectively in patients with SMA. The authors of the 6MWT and the rNHPT use similar methodology in which subjects are instructed to deliver maximal performance and change in velocity or distance is assessed as primary outcome measure. The simple instruction and relatively short test period (1.5–6 min) make them particularly useful to detect fatigability in the individual patient with SMA. There were, however, several intrinsic clinical properties of both tests which made them less appropriate to assess the construct of fatigability as defined in this study: The intensity was not standardized and might fluctuate between maximal and submaximal intensity within and between subjects depending on disease severity and motivation [42, 43]; The change in velocity as primary outcome measure did not directly reflect the inability to sustain prolonged repetitive task during ADLs such as frequently reported by patients; Both tests did not cover the subgroup of non-ambulatory patients with antigravity function of the arms, who primarily experience problems with repetitively lifting the arm while drinking or eating. The expert panel discussed whether potential non-validated outcome measure for fatigability were available that were more standardized on performance and used meaningful outcome parameters for endurance capacity. The methodology of the Endurance Shuttle Walk Test (ESWT) was proposed by one of the experts with experience in chronic pulmonary disease.

### The endurance shuttle walk test

Revill et al. developed a externally controlled constant paced walking test to assess endurance capacity in patients with chronic obstructive pulmonary disease [44] (Additional file 1). The expert panel judged the methodology of ESWT as superior to all other outcome measures with regards to external regulation of pace, test duration and standardization of intensity. Since the ESWT could only be used in ambulatory patients, it was concluded that alternative outcome measures using the same methodology should be ideally used for endurance testing in non-ambulatory patients. Since no such outcome measure were available, it was decided to select existing scales that corresponded well with reported activities by patients and incorporate them in to the methodology of the ESWT.

### Selecting and formulating items

Patients with SMA reported a great number of different activities on the domains of leg function, upper arm function and hand function (Table 3). The expert panel therefore decided that all domains should be included in the development of the set of outcome measures for fatigability. The ESWT was selected to cover the activities related to leg muscles. The upper arm domain mainly comprehended activities lifting an object while the hand function domain mainly included activities performed at the table while moving around the lower arm and hand. To cover activities of the upper arm and hand function, the expert panel decided to apply the methodology of the ESWT to the Nine Hole Peg Test and the Box and Block Test resulting in the Endurance Shuttle Nine Hole Peg test (ESNHPT) and the Endurance Shuttle Box and Block Test (ESBBT). The Nine Hole Peg Test, originally developed to assess distal arm function demonstrated good feasibility and sensitivity to detect fatigability in patients with SMA type 2 [41, 45, 46]. The Box and Block Test, a measure for upper limb motor function, represented antigravity activities of the arms such as brushing teeth, eating a sandwich and lifting a cup [47, 48].

**Table 3:** Daily life activities provoking fatigability clustered per functional domain

Leg function	Proximal arm function	Hand function
*Walking*	*Lifting a cup*	*Writing*
*Climbing stairs*	*Brushing teeth*	*Eating a sandwich*
*Cycling*	*Throwing a ball*	*Typing*
*Swimming*	*Fishing*	*Riding a power driven wheelchair*
*Showering*	*Holding phone to ear*	*Cutting (scissors)*
*Playing soccer*	*Washing hair*	*Drawing*
*Running*	*Carrying a bag*	*Painting*
*Putting clothes in the washing machine*	*Shoe polishing*	*Playstation*
	*Dish washing*	*Using cutlery*
	*Using cutlery*	*Driving car with mini joystick*
	*Showering*	*Moving things on the table*
	*Cooking*	*Using Mousepad*
	*Vacuum cleaning*	*Taking money out of wallet*
	*Washing clothes*	*Clapping hands*
	*Riding a hand driven wheelchair*	*Fixing screws*
	*Swimming*	*Putting on make-up*
	*Hanging clothes to dry*	*Moving objects on wheelchair table*

### Scoring issues

In accordance with the original ESWT, Time to Limitation (Tlim (sec)) was chosen by the expert panel as the primary outcome measure of all three endurance tests (round table discussion 3). With the aim to eventually use the set of tests both in research and clinical practise time constraints in the latter had to be taken in account [17]. To improve both motivation for and feasibility of tests, maximum test duration was shortened from 20 min to 10 min. Based on clinical experience, it was expected that 10 min would be a sufficient time period to measure fatigability.

### Pilot testing

#### Pilot-test sample 1 and 2

Eight healthy adults and one adolescent (mean age = 28.7, 50% female) performed all three endurance tests. Respectively 30 and 44% of the subjects could not continue at an intensity level of 85% for at least 10 min during the ESWT and ESBBT. Early termination was primarily caused by subtle coordinative errors due to the high velocity at which the motor task was performed. Therefore, intensity level of 85% was not considered valid for the assessment of fatigability in patients with SMA. Assessment at a 65% intensity level was considered too easy. It was therefore decided to set the intensity level at 75% for all tests. Consecutively a second pilot study was performed in 10 children with neuromuscular diseases and other motor disabilities (Developmental Coordination Disorder (*N* = 1), Cerebral Palsy (*N* = 2), SMA (N = 2), Duchenne Muscular Dystrophy (N = 2), Spina Bifida (N = 1), Acquired Brain Injury (N = 1) and Spinal Cord Injury (N = 1)) to determine the feasibility of the endurance tests. All participants showed good comprehensibility and acceptability of the tests without any adverse events. Three children (SMA (*n* = 2), Spinal Cord Injury (*n* = 1) demonstrated a decreased time to limitation.

#### Pilot-test sample 3

Fifteen patients with SMA type 2 (*n* = 8), type 3a (*n* = 5) and type 3b (*n* = 3) aged 10–49 and with a broad range in clinical severity (Hammersmith Functional Motor Scale Expanded score = 0–66) (Table 4) performed 1,2 or 3 of the endurance shuttle tests (i.e. ESNHPT, ESBBT, ESWT) tests depending on their level of motor function. The comprehensibility, acceptability and measurement completion of all three tests were excellent despite moderate to severe self-reported muscle fatigue. All subjects were strongly motivated to perform well on the test and willing to do the test again in the context of future studies. Beforehand, it was expected that at least 50% of the subjects would end the test prematurely because of fatigability. Although most subjects did show signs of fatigability at the end of the test reflected by decrease in coordination, compensatory movements and perceived exertion, the drop –out rate was lower than expected on the ESNHPT (31%), ESBBT (45%) and ESWT (50%). The ESWT showed a trend towards ceiling effect (Tlim (Mn) = 462/600 s). It was observed that during the ESBBT subjects were actively compensating for fatigability by leaning on the box.

**Table 4:** Pilot sample 3

	ESWT	ESBBT	ESNHPT
Sample size	4	9	13
SMA type			
2	0	3	6
3a	1	3	4
3b	3	3	3
Age			
yrs (min.-max.)	26.2 (10–37)	20.8 (10–37)	23.9 (10–49)
Gender			
Male	2	6	8
Female	1	3	5
HFMSE			
0–66	52 (44–66)	31 (4–66)	22 (1–66)
Time to Limitation			
0–600	555 (462–600)	373 (83–600)	457 (52–600)
Reduced time to limitation			
Yes	50%	44,4%	30,8%
No	50%	55,6%	69,2%
Measurement completion			
Yes	100%	100%	100%
No	0%	0%	0%
Comprehensibility			
Yes	100%	100%	100%
No	0%	0%	0%
Acceptability			
0–10 (min. – max.)	9.2 (7.4–10)	9.6 (7.9–10)	8.9 (4.9–10)
Perceived burden			
Muscle fatigue	7 (6–9)	4.9 (3–9)	4.5 (1–10)

*ESWT* = Endurance Shuttle Walk Test, *ESBBT* = Endurance Shuttle Box and Block Test, *ESNHPT* = Endurance Shuttle Nine Hole Peg Test

### The endurance shuttle tests: Materials and procedures

To improve the validity of the tests, the protocol was modified on two important aspects. First, the maximal test duration was lengthened to 20 min for all tests (in accordance with the original ESWT procedure). Second, we decided that compensatory movements (e.g. leaning on the box during the performance of the box and block test) was no longer allowed. Materials and procedures for the set of endurance tests were described (Additional file 2).

## Discussion

This study aimed to provide the framework for the development of novel clinical outcome measures for fatigability in patients with SMA across the range of severity. The major strength of this study includes the use of the methodological steps as recommended by the COSMIN guidelines to systematically develop a set of endurance tests for patients with SMA with a specific emphasis on content validity [17]. Content validity is the degree to which the content of an instrument is an adequate reflection of the construct to be measured and without it, it is difficult to select appropriate outcome measures for trials or other types of interventions [49]. It is therefore recommended by the US Food and Drug Administration and the European Medicines Agency to establish content validity before evaluating other measurement properties [15, 16]. The content validity of the endurance shuttle tests was established by combining evidence from scientific literature with patient reported outcome and the expertise from health care professionals and scientists, which will potentially lead to both valid and clinically meaningful outcome measures.

An important aim of this study was to develop one methodology for a broad clinical spectrum that would enable comparison between severely and mildly affected patients and with that facilitate future study trial inclusion. The methodology of the ESWT, originally validated for pulmonary disease was adjusted and applied to other motor tasks to meet with the specific disease characteristics of SMA. The ESWT speed was originally derived from a time consuming four component process including a second ISWT and a regression equation including maximal predicted oxygen uptake. Although Hill et al. simplified this method by directly using maximal walking speed it still included a second exercise test [50]. We questioned the validity of this method because of the risk of inducing fatigability prior to the test and therefore decided to use muscle power as the parameter to determine exercise intensity in SMA. Time in which 10 m, 9 pegs or 10 blocks could be transferred were taken as maximal performance measure. It was decided not to adjust for the weight of the blocks and pegs or body weight, since both materials were very light and body weight is fixed in daily life activities as well. The convergent validity of this modified method with the original procedures and the comparability between patients with mild and severe muscle weakness need to be further analysed in future studies.

We decided to include motor tasks because we wanted to generate clinical relevant outcome measures and patients with SMA generally have normal coordinative function. The use of motor task within endurance tests potentially causes validity issues. For example, a subject might drop out because of motor coordination difficulties rather than fatigability. To confirm construct validity, it will be important to monitor other parameters of fatigability such as perceived exertion, motor behaviour and change in strength and electromyography response [38, 40, 51, 52].

Besides the clinimetric properties, the practical application of a new measurement test is an important aspect in the development of outcome measures for clinical practice. Ideally, an outcome measure is suitable for both day-to-day clinic purposes and clinical trials. For this purpose, an instrument needs to be easy to use in a limited time period, acceptable and feasible for the individual subject, while at the same time, applicable to a large part of the study population. The endurance tests have demonstrated to be comprehensible and acceptable for both healthy subjects and patients with a wide range of severity in an age range of 10–49 years. Based on our clinical experience and an upcoming large study on validity and reliability (Bartels et al. in progress), we expect the endurance tests to be suitable for subjects aged 6 years and older for those being able to move around their dominant hand on their wheelchair table as minimal motor function. The additional burden and time consumption in the context of endurance tests as part of the already extensive trial assessments asks for a clear rationale about the efficacy in terms of function accompanied by the selection of the most appropriate tests. In order to be able to measure clinically relevant improvement in endurance, endurance tests that mimic long-term activities are required.

In the current literature, the concept of fatigability and fatigue are often used interchangeably with different terms such as fatigue [25], fatigability [7], neuromuscular fatigue [53], perceived fatigability [54], physiological fatigue [55], physiological fatigability [19], physical fatigue [20], peripheral fatigue [22], muscle fatigue [21, 56, 57] and so on. The lack of standard definitions and the inconsistency of terminology hamper the advancement in our understanding of the pathophysiological background of fatigability in SMA and the development of appropriate outcome measures. The taxonomy used in this study was particularly suitable to standardize definitions and clarify the different concepts and means of measurements as a prelude to the development of an outcome measure for fatigability in SMA. The taxonomy makes an important distinction between ´perception´ and ´performance´, which are measured at a different level. Perception of fatigue is defined as the subjective sensations of weariness, increasing sense of effort, mismatch between effort expended and actual performance or exhaustion while fatigability is about decline in either physical or mental performance. Although there is a clear distinction in definitions and means of measure, endurance performance is regulated by an interaction between fatigability and perceptions of fatigue and influenced by physchological factors, peripheral limitations and central factors [6, 57–59]. Therefore, a psychophysiological approach is needed when interpreting the outcome of endurance testing in patients with SMA. Based on both pre-clinical and clinical data it was hypothesised that fatigability would be associated with neuromuscular junction dysfunction in at least half of the patients with SMA and therefore, similar to the approach in myasthenic syndromes, best provoked with a repetitive submaximal prolonged motor task. Although Spinal Muscular Atrophy is primary characterised by loss of motor neurons, involvement of other systems such as autonomic dysfunction and altered muscle metabolism is reported and might demand additional methods of testing to capture fatigability in SMA [60, 61]. Fatigability in SMA could also be related to an increased energy cost of movement due to progressive muscle weakness and secondary deconditioning [20, 23]. Therefore, the individual disease course and physical activity levels should be taken in account when measuring fatigability and its change in time.

### Limitations

We decided to limit our search for existing outcome measures to a scoping review in SMA although a systematic review in the entire range of neuromuscular diseases could have generated other endurance measures. Based on experience by the expert panel and the specific characteristics of SMA regarding clinical variability and complaints of fatigability, it was anticipated that a time consuming systematic review would give very limited additional information as hardly any endurance testing has been developed in neuromuscular diseases. The involvement of patients in rare disease clinical trial design is increasingly becoming a priority [62]. Although established methods such as face-to-face meeting and focus groups were not applied yet, extensive questionnaires provided a valuable insight in the patient perspective on fatigability. In the further development of the endurance tests, patients will continue to play an important role.

## Conclusions

Fatigability has emerged as an important dimension of physical impairment in patients with SMA. The development of a comprehensive set of endurance tests is a pivotal next step to facilitate intervention studies on fatigability and address this important complaint in patients with SMA. We developed a set of endurance tests for both non-ambulatory and ambulatory children and adults with SMA which meet predefined specific criteria to achieve three main objectives: 1) quantify endurance; 2) generate clinical relevant outcome parameters and; 3) cover a large part of the clinical spectrum of SMA. Reliability and construct validity need to be investigated in future studies.

## Additional files

Additional file 1: Description of outcome measures considered for selection. *Description of data:* Description of studies about outcome measures considered for selection. (DOCX 17 kb)

Additional file 2: The Endurance Shuttle Tests: materials and procedures. *Description of data:* description of the materials and procedures needed to perform the endurance tests. (DOCX 334 kb)

## Abbreviations

6MWT: Six minute walk test
ADLs: Activities of daily living
COSMIN: COnsensus-based Standards for the selection of health Measurement INstruments
ESBBT: Endurance shuttle box and block test
ESNHPT: Endurance shuttle nine hole peg test
ESWT: Endurance shuttle walk test
ISWT: Incremental shuttle walk test
r9HPT: repeated nine hole peg test
SMA: Spinal muscular atrophy
Tlim: Time to limitation
