# Supplementary material for: Assessment of fatigability in patients with spinal muscular atrophy: development and content validity of a set of endurance tests
Source: BMC Neurol. 2019 Feb 9;19:21. doi: 10.1186/s12883-019-1244-3 (PMC6368708; doi:10.1186/s12883-019-1244-3)
Supplement: Supplementary file 1 — Description of outcome measures considered for selection. Description of data: Description of studies about outcome measures considered for selection. (DOCX 17 kb) [file 12883_2019_1244_MOESM1_ESM.docx]

**Additional file 1: Description of outcome measures considered for selection**

Sustained maximal voluntary contraction for 60 seconds Milner-Brown studied fatigability of knee extensors and ankle dorsiflexors in 15 patients with neurogenic muscle weakness including five patients with SMA aged 16-55 years and 20 healthy controls aged 18-55 years. Subjects were asked to exert maximum force against a electromechanical device incorporating a force transducer and maintain maximum effort for one minute. Fatigue Index (FI) was expressed as the percentage decrease in maximal force at the end of 60 seconds. The mean FI of both ankle dorsiflexion (50 % ± 15 versus 34% ± 13) and knee extensors (62% ± 17 versus 46% ± 15%) of patients was significantly greater than in controls (p < 0.01) and characterised by a steep decrease in performance by patients from 30 to 60 seconds.

Sustained maximal voluntary contraction for 15 seconds Iannacone et al. studied fatigability of knee flexors, knee extensors, elbow flexors and elbow extensors in 72 ambulatory and non- ambulatory patients with SMA aged 5-57 years and 24 healthy controls aged 5-32 years. Subjects were asked to push or pull against a fixed myometer as hard as possible and hold for 15 seconds, while given audio feedback. The maximal voluntary contraction times 15 seconds represented 100% of endurance or no fatigability. Endurance was expressed as the area under the curve for each maximal voluntary contraction. The authors found a large variability in endurance (AUC of 50-90%) with a similar response in patient and controls.

Masticatory endurance Granger et al. studied masticatory muscle endurance in 15 patients with juvenile onset SMA aged 6-20 years and 15 age- and sex-matched healthy controls. Subjects were asked to hold a 60% sub-maximum bite force level for as long as possible while being timed. Patients with SMA (11.1 seconds) fatigued faster than controls (17.9 seconds) (p = 0.03).

The six minute walk test (6MWT) Montes et al. studied fatigability during ambulation in patients with SMA type 3 aged 4-49 year in 4 separate studies. Subjects were instructed to walk as far as possible along a 25-m course during 6 minutes. Encouragements during the test were standardized according to the American Thoracic Society (ATS) – guidelines. Distance walked each minute and time to complete each 25-m segment were recorded. Montes et al. found a range of 11-21% decrease in walking distance between the 6^th^ and the 1^st^ minute.

The Repeated Nine Hole Peg Test (r9HPT) Stam et al. studied fatigability of the arm and hand in fifty two patients aged 7-72 years with SMA type 2-4, 17 healthy aged 6-73 years and 29 disease controls aged 8-76 years. Subjects were asked to perform five consecutive rounds of the Nine-Hole Peg Test as fast as possible without a break ([41](#_ENREF_41)). The time required to complete each round was recorded and compared to the first round. Time needed to complete each round during the five-round task increased in 65% of patients with SMA type 2, 36% of type 3a, 22% of type 3b/4, 31% of disease controls and 6% of healthy controls. Patients with SMA type 2 performed the test significantly more slowly (+27%) than all other groups (p<0.005). This study was published recently and given the promising results, the r9HPT was included in the assessment of potential outcome measures

The Endurance Shuttle Walk Test Subjects walked at 85% intensity, derived from the walking speed at 85% of peak VO_2_ uptake during a separate Incremental Shuttle Walk Test (ISWT). Subject were instructed to continue walking on a 10 meters shuttle course until too tired or breathless to continue with a cut off time of 20 minutes. Subjects were given no indication of how long they were walking and were not informed of the 20 minutes limit. Walking speed was externally regulated by a beep signal and the test was terminated prematurely when subjects failed two times in a row to reach the other side within time. The ESWT demonstrated good test-retest reliability and sensitivity to change after a seven week rehabilitation program.
