# Supplementary material for: Assessment of fatigability in patients with spinal muscular atrophy: development and content validity of a set of endurance tests
Source: BMC Neurol. 2019 Feb 9;19:21. doi: 10.1186/s12883-019-1244-3 (PMC6368708; doi:10.1186/s12883-019-1244-3)
Supplement: Supplementary file 2 — The Endurance Shuttle Tests: materials and procedures. Description of data: description of the materials and procedures needed to perform the endurance tests. (DOCX 334 kb) [file 12883_2019_1244_MOESM2_ESM.docx]

**Additional file 2: The Endurance Shuttle Tests: materials and procedures**

|  | **ESWT** | **ESBBT** | **ESNHPT** |
| --- | --- | --- | --- |
|  | 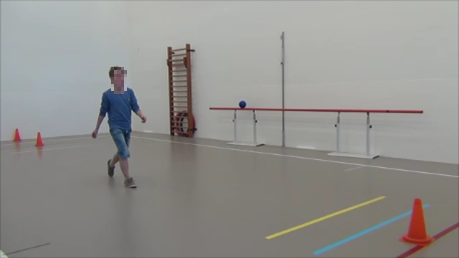 | 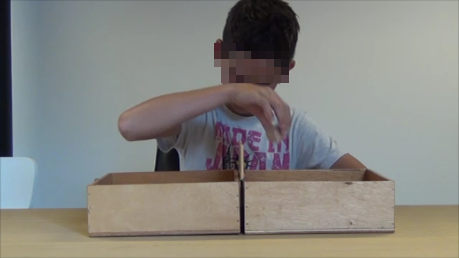 | 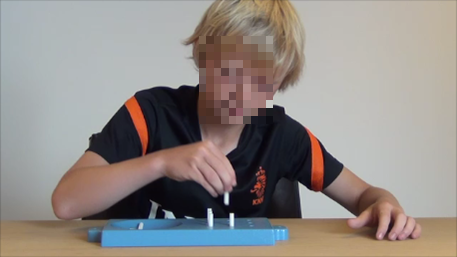 |
| **Test Material** | - Straight corridor - 10 meter walking course - 4 cones - Metronome | - Box and Block Test - 200 blocks - Adjustable table - Metronome | - Nine Hole Peg Test - Adjustable table - Metronome |
| **Maximal performance estimation** | - Walk as fast as possible and turn at the line between the cones before the beep - 5-10 trials, 30 seconds breaks - Fastest time out of three attempts < 10% difference | - Transfer 10 blocks over the partition as fast as possible before the beep - 5-10 trials, 30 seconds breaks - Fastest time out of three attempts < 10% difference | - Place and return the nine pegs as fast as possible before the beep - 5-10 trials, 30 seconds breaks - Fastest time out of three attempts < 10% difference |
| **Intensity level** | - 75% individual intensity (s) = maximal time(s)/0.75 and then converted into the matching metronome number | - 75% individual intensity (s) = maximal time(s)/0.75 and then converted into the matching metronome number | - 75% individual intensity (s) = maximal time(s)/0.75 and then converted into the matching metronome number |
| **Maximal duration** | - 20 minutes | - 20 minutes | - 20 minutes |
| **Instruction Assessor** | - Cover each time 10 meters before the beep - Continue as long as possible within safety margins - Try to speed up in case of one failure | - Transport each time 10 blocks before the beep - Continue as long as possible - Try to speed up in case of one failure | - Place and return 9 pegs each time before the beep - Continue as long as possible - Try to speed up in case of one failure |
